# The effect of physical exercise on disordered social communication in individuals with autism Spectrum disorder: a systematic review and meta-analysis of randomized controlled trials

**DOI:** 10.3389/fped.2023.1193648

**Published:** 2023-06-30

**Authors:** Shuqi Jia, Chengcheng Guo, Shufan Li, Xiaojing Zhou, Xing Wang, Qiang Wang

**Affiliations:** ^1^ School of Physical Education, Shanghai University of Sport, Shanghai, China; ^2^ School of Public Administration, Hohai University, Nanjing, China; ^3^ School of Physical Education and Health, Shanghai Lixin University of Accounting and Finance, Shanghai, China

**Keywords:** physical activity, exercise, autism spectrum disorder, social communication disorder, meta-analysis

## Abstract

**Objective:**

The aim of this systematic review and meta-analysis was to systematically investigate the intervention effect of physical exercise on disordered social communication in patients with autism spectrum disorders.

**Methods:**

This systematic review and meta-analysis used the PubMed, Web of Science, the Cochrane Library, and Embase electronic databases to conduct a systematic search of literature describing randomized controlled trials (RCTs) on the effect of physical exercise on disordered social communication in autistic patients from the first year of inclusion to 21 January 2023.

**Results:**

A total of 14 RCTs including 460 autistic patients were analyzed. A meta-analysis showed that physical exercise had a positive effect on social communication disorder (SMD = 0.45, 95% CI: 0.15, 0.74, *p* < 0.05) in autistic patients. Subgroup analysis showed that exercise programs with multiple components (SMD = 0.73, 95% CI: 0.39, 1.06, *P* < 0.001), a moderate duration (SMD = 0.73, 95% CI: 0.38, 1.08, *P* < 0.001), a moderate-high frequency (SMD = 0.84, 95% CI: 0.53, 1.14, *P* < 0.001), and a long duration (SMD = 0.77, 95% CI: 0.36, 1.18, *P* < 0.001) led to significant improvement.

**Conclusion:**

Physical exercise can improve disordered social communication in patients with autism spectrum disorders. Specifically, early intervention, multi-component exercise, a moderate period, moderate and high frequency, long duration, and multi-participant programs were most effective.

**Systematic Review Registration:**

https://www.crd.york.ac.uk/prospero/ RecordID= CRD42023422482.

## 1. Introduction

Autism Spectrum Disorder (ASD) is a group of early-onset neurodevelopmental disorders associated with altered social communication and interaction, with repetitive stereotyped interest behaviors as core symptoms. Social communication disorder, characterized by persistent social communication and interaction deficits, is a core feature of ASD (1). These abnormal patterns can affect the development of social skills, preventing children from effectively participating in multi-context social interactions in the home, school, and community, with serious implications for survival and growth (2). In recent years, the global incidence of ASD has been rising. The prevalence of ASD in developed countries is approximately 1.5% (3), and according to the US Centers for Disease Control and Prevention, it was 2.47% in children aged 0–8 years in 2021 (4, 5). At present, there is no effective drug to treat ASD, and the function of existing drugs is limited to delaying the progression of the condition and alleviating the onset of social communication disorder in some autistic patients (6, 7).

Physical exercise has increasingly been shown to improve core symptoms in patients with ASD (8, 9). Individuals with ASD can participate in physical activity individually or in groups, and through watching and imitating their peers, they can learn social etiquette and social communication, and improve their visual processing ability and attention. This can ultimately improve cognitive function, and positively affect brain activation (10, 11). Physical exercise can resemble stereotypical behaviors in individuals with ASD. Targeted activities can replace non-targeted stereotypical actions such that patients no longer need to experience pleasure from stereotypical behaviors (12). When playing ball games, the inevitable communication behavior between children and their peers or parents can encourage children to engage in social activities. Similar findings have been reported for karate training (13). Children with ASD learn social communication by watching their peers, which emphasizes the importance of visual processing. In both laboratory and real-life social scenarios, an inability to establish eye contact with others severely affects communication in children with ASD. Sensory integration training can help improve visual processing skills in children with ASD and enhance their social processing abilities, thus promoting their social interaction skills (14). Basic social etiquette is also included in sports training, such as greeting peers, parents, or teachers during basketball training, queuing, etc., which increases communication, physical contact, eye contact, language, and promotes social behavior in children with ASD (15).

In terms of social interaction, physical exercise is considered a safe and effective treatment option for improving core symptoms in patients with ASDs. Currently, there are no objective, effective, and specific early diagnostic biomarkers or drugs for the social problems associated with ASD, and medical needs are still not being met (16, 17). There are currently many treatment options, but the treatment effects of different rehabilitation methods vary, and there are associated problems such as high economic costs and low practicability. For example, language therapy, occupational therapy, physical therapy, and behavioral interventions may represent a heavy economic burden for individual families and social service agencies (18). Physical exercise has few side effects, low economic cost, and is easy to implement. Although physical activity does not address the core causes of this disorder, increasing levels of physical activity may provide more opportunities for social interaction with peers, better attention and motor performance, and thus indirectly impact the core symptoms of ASD (19).

Previous research has shown differences in the effectiveness of physical exercise interventions for patients with ASDs. Research has found through a meta-analysis that the effect of organized physical activity on communication in individuals with ASD was not statistically significant, and that there were only small and medium improvements in total social interaction (20); a study revealed detected a low correlation between overall exercise and social skills, although more experiments are required to support the improvement effect of exercise in autistic patients (21). There are many reasons for inconsistencies in the research findings, including the diversity of exercise forms and the great distinctions among physical exercise variables (intensity, duration, frequency, and period) designed by researchers (22).

Few meta-analyses have examined the intervention effects of physical exercise on social communication function in patients with ASD, and most studies have not examined the “dose-response” relationship between physical exercise variables and social communication difficulties. Therefore, this study addressed the following research questions: “Can physical exercise improve social interaction problems?” “What dosages of physical exercise can best improve social interaction problems?”. This study employed a meta-analysis of published randomized controlled trials (RCT) studies, with social communication difficulties in patients with ASD as the outcome measure, and conducted subgroup analyses of factors such as type of exercise, exercise duration, exercise frequency, exercise intensity, and exercise timing, with the aim of exploring the best exercise program based on these factors and providing evidence-based support for future research in this field.

## 2. Methods

This systematic review was prospectively registered with the National Institute for Health Research website PROSPERO. Details of the protocol can be accessed at: https://www.crd.york.ac.uk/prospero/RecordID=CRD42023422482.

### 2.1. Search strategy

The research followed the PRISMA Statement (Preferred Reporting Items for Systematic Reviews and Meta-analysis) and the Cochrane Workbook, and the included literature was collated and counted according to the requirements of the International Systematic Review Writing Guidelines (23).

This study used the PubMed, Web of Science, the Cochrane Library, and Embase electronic databases to conduct a systematic search of literature describing randomized controlled trials (RCTs) on the effect of physical exercise on disordered social communication in autistic patients from the first year of inclusion to 21 January 2023. used the PubMed, Web of Science, Embase, and Cochrane Library electronic databases to search for Chinese and English studies published both domestically and overseas. The search period was from the database's creation until 21 January 2023. Using combinations of subject words and free words, the following search terms in English were used: [“Movement” OR “Physical exercise” OR “Physical activity” OR “Exercise” OR “Sport” OR “Training, Exercise” OR “Physical Exercises” OR “Training” OR “motion” OR “activity” OR “physical therapy”] AND [“autistic disorder” OR “Autism Spectrum Disorders” OR “Autistic Spectrum Disorders” OR “Disorder, Autistic” OR “Spectrum” OR “Early Infantile Autism” OR “Disorders, Asperger” OR “Syndrome, Asperger”] AND [“randomized controlled trial” AND “randomized” OR “controlled” OR “trial” OR “random” OR “random allocation” OR “RCT” OR “RCTs”]. Meanwhile, the references of the included literature and related reviews were traced back to calculate the recall ratio.

### 2.2. Inclusion criteria

(1) Study type: RCTs; (2) Study population: Individuals diagnosed with ASD or individuals with a diagnosis of ASD who met the diagnostic criteria of the American Psychiatric Association's Diagnostic and Statistical Manual of Mental Disorders, Fourth Edition (DSM-IV) or Fifth Edition (DSM-Ⅴ); (3) Intervention: The control group received routine rehabilitation treatment or no intervention, while the experimental group received sports training in addition to routine rehabilitation treatment; (4) Outcome measures: The primary or partially primary outcome measures were social interaction and social skills.

### 2.3. Exclusion criteria

(1) Reviews, comments, animal experiments, and duplicate publications; (2) Studies with unclear descriptions of experimental data, incomplete data, an inability to obtain raw data even after contacting the authors, or poor quality; (3) Studies involving subjects with other physical diseases; (4) Studies involving animal-assisted therapy in the intervention group; (5) Studies with unclear diagnostic criteria or intervention plans.

### 2.4. Data extraction

Two researchers independently screened the literature and extracted the data, then cross-checked the data. Any disagreements were discussed with a third researcher. The data extraction mainly included: basic information about the studies (first author, year of publication, and nationality of author), fundamental information about the experimental subjects (sample size, age, and gender), physical exercise variables (intervention, period, frequency, duration, and organizational form) and outcome indicators, as shown in Table 1.

**Table 1:** Intervention characteristics of included studies.

Study	Country	Sample size (E/C)	Mean age/years (E/C)		Intervention type		Intervention dose			Organizational form	Outcome indicator
			Experimental group	Control group	Experimental group	Control group	Period (week)	Frequency (times/week)	Duration (minute/times)		
Agnes S. Chan2013 (24)	China	20/20	11.28 ± 3.90	12.42 ± 3.25	Nei Yang Gong	Progressive Muscle Relaxation	4	2	60	Ⅰ	ATEC
Ahmadreza Movahedi2013 (13)	Iran	13/13	9.54 ± 3.43	9.06 ± 3.33	Karate	Daily activities	14	4	90	Ⅰ	GARS-2
Fatimah Bahrami 2015 (25)	Iran	15/15	9.20 ± 3.32	9.06 ± 3.33	Karate	No arrangements	14	4	90	Ⅰ	GARS-2
Janice N. Phung2019 (26)	The United States	14/20	9.10 ± 1.10	9.52 ± 1.07	Mixed martial arts/Martial arts intervention	Daily activities	13	2	45	Ⅰ	SSIS
Jin-Gui Wang2020 (15)	China	18/15	5.11 ± 0.65	4.70 ± 0.70	Mini-Basketball Training	Daily activities	12	5	40	Ⅱ	SRS-2
Kelong Cai2020 (27)	China	15/14	5.13 ± 0.61	4.68 ± 0.72	Mini-Basketball Training	daily activities	12	5	40	Ⅱ	SRS-2
Ke-Long Cai2020 (10)	China	15/15	4.56 ± 0.84	5.03 ± 0.64	Mini-Basketball Training	Daily activities	12	5	60	Ⅱ	SRS-2
Mirella Zanobini2019 (28)	Italy	13/12	5.68 ± 1.02	5.42 ± 1.54	Swim	Conventional treatment	24	0.5	30	Ⅲ	SRS-2
Sabine C Koch2015 (29)	Germany	16/15	22 + 7.7		Dance Movement	No arrangements	7	1	60	Ⅱ	FBT
Sixin Yang2021 (30)	China	15/15	5.03 ± 0.55	4.67 ± 0.70	Mini-Basketball Training	Conventional rehabilitation	12	5	40	Ⅱ	SRS-2
Wenxin Xu2019 (14)	China	50/53	6.17 ± 2.44	6.18 ± 2.94	Sensory Integration Training	Conventional treatment.	12	NR	NR	Ⅱ	ABC
Mahboubeh G 2018 (31)	Iran	12/14	7.08 ± 2.06	8.07 ± 2.23	Sports games	Conventional treatment.	12	3	40	Ⅲ	GARS-2
Amir H.H2022 (32)	Iran	8/8	6∼10		Comprehensive Physical Training	Conventional treatment.	8	3	60	Ⅱ	GARS-2
Supritha Aithal2021 (33)	Britain	10/16	11.53	9.77	Dance Movement	Daily activities	5	2	40	Ⅱ	SCQ

For convenience, only the first author is given. NR means not reported, and E/C means experimental group/control group. Individual exercise with group participation refers to individual exercise where group activities accounted for more than 50% of the total duration. (I: individual exercise; II: group exercise; III: individual exercise with group participation).

### 2.5. Quality assessment

The PEDro scale (Physiotherapy Evidence Database) was used to rate the quality of the RCTs. The calibration of the scale was established *via* the Delphi scale, and two calibrations were conducted. The scale included 11 evaluation items: “eligibility criteria”, “random allocation”, “concealed allocation”, “similar baseline”, “blinding of subjects”, “blinding of therapists”, “blinding of outcome assessment”, “dropout rate ≤ 15%”, “intention analysis”, “between-group statistical comparisons”, and “point measures and measures of variability”. Among them, “eligibility criteria” was not counted in the scoring system. If a certain criterion was explicitly met, it was given a score of 1, otherwise, it received a score of 0. The total score was 10, with “<4” indicating poor quality, “4–5” medium quality, “6–8” good quality, and “9–10” high quality. Two authors independently graded the quality of the included studies, and if there was a disagreement, they discussed it with the third author until there was a consensus.

### 2.6. Statistical analysis

Reviewer Manager 5.4 was used for effect size pooling, subgroup analysis, heterogeneity testing, and sensitivity analysis. Stata 17.0 was used for publication bias testing. The outcomes included in this review were all continuous variables, with the mean difference (MD) and standardized mean difference (SMD) shown. If there were no significant differences in the baseline, the endpoint value (mean standard deviation, M ± SD) of the experimental group and the control group after the intervention were used as the main effect parameter. If the literature only provided the change value, it was estimated using the Review Manager 5.4 algorithm. Heterogeneity testing was performed using *p* and *I^2^*, and the fixed effects model was used for meta-analyses if there was no statistical heterogeneity among the research results (*I^2 ^*< 50%, *p *> 0.10). Otherwise, the random effects model was used and the reasons for the heterogeneity were analyzed. If there was clinical heterogeneity, subgroup analysis or sensitivity analysis were conducted according to its source. If there was no obvious clinical heterogeneity, the random effects model was used for the meta-analysis. If the heterogeneity was too large or the indicators could not be merged, descriptive analysis was performed.

## 3. Results

### 3.1. Search results

**Figure 1:**
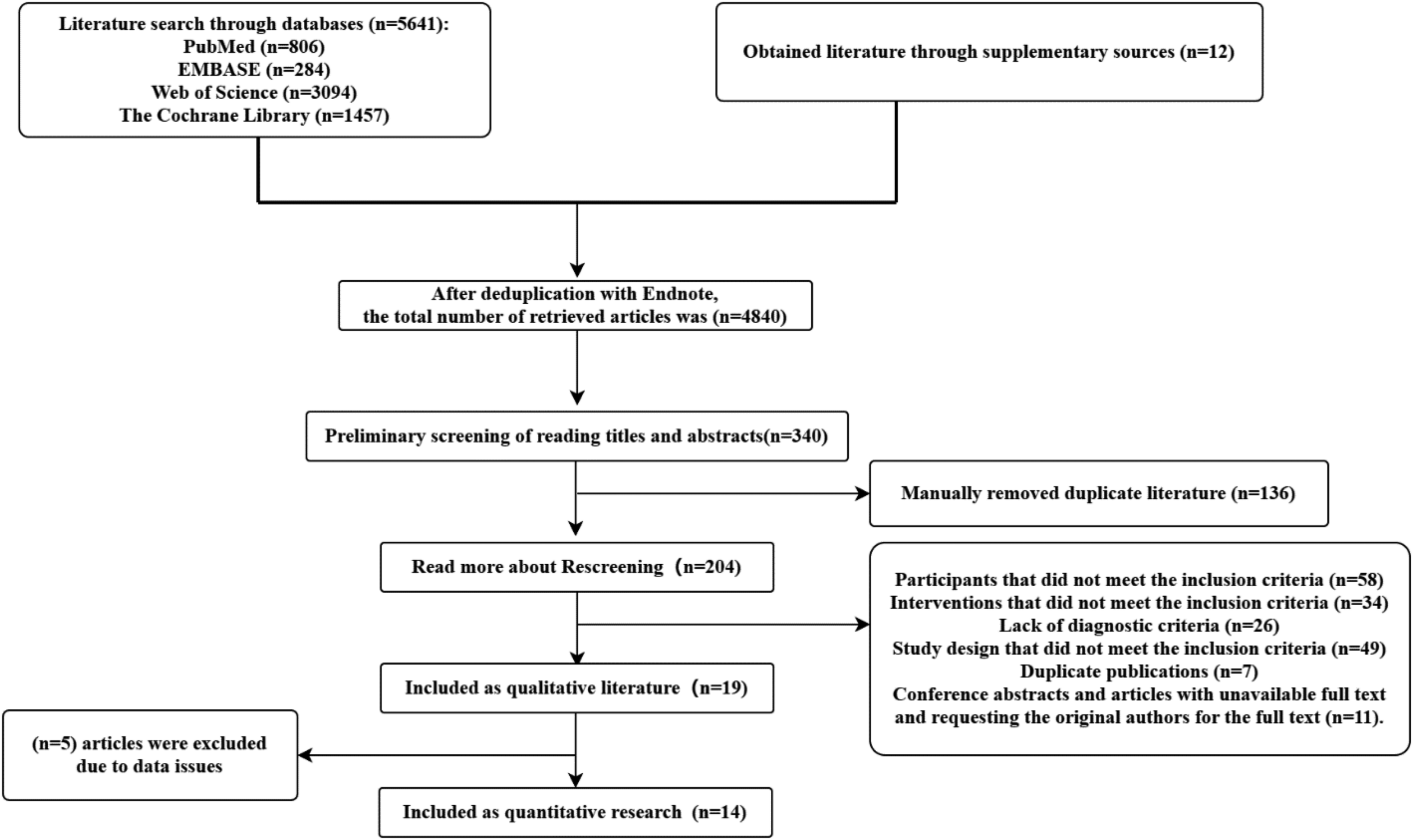
Flow diagram of study screening.

A total of 6,678 studies were retrieved by searching the PubMed, WOS, Cochrane Library, and Embase databases, and 12 were manually retrieved. These studies were imported into EndnoteX9 to conduct statistics and collation, where 5,877 were obtained after duplication removal, 340 were obtained after preliminary screenings of topics and abstracts, and 16 were finally included in the quantitative analysis after reading the full text (Figure 1).

### 3.2. Basic information and intervention characteristics of the included studies

There were 14 studies (*N* = 460) in the included literature (Table 1) published from 2013 to 2022. The participants were all patients with ASD, and the sample sizes of all the studies included in the analysis were selected according to the outcome indicators. There were no significant differences in baseline values. Among the included studies, six were from China, four were from Iran, and the remaining four were from the United States, Britain, Italy, and Germany.

Table 1 shows that all included studies reported all or some of the physical exercise variables, providing the specific intervention form, exercise period, exercise duration, exercise frequency, organizational form, and intervention methods for the control group. There were three different types of physical exercise, such that five papers examined aerobic exercise (35.7%), six used mind-body exercise (42.9%), and three used multi-component exercise combining various exercises (21.4%).

### 3.3. Quality evaluation of included studies

As shown in Table 2, all 14 included RCTs achieved a “similar baseline”, “intention analysis”, “between-group statistical comparisons”, and “point measures and measures of variability”. Among them, two papers achieved “concealed allocation” (24, 28), two used “blinding of therapists” (25, 28), three achieved “blinding of outcome assessment”(13, 28, 30), and one did not fulfill “dropout rate ≤ 15%”(29). In terms of the PEDro score, one paper scored 4–5, 12 scored 6–8, one scored 9–10, and the average score was 6.43. Overall, the methodological quality was good.

**Table 2:** Methodological quality of the included studies.

Included study	Random allocation	Concealed allocation	Similar baseline	Blinding of subjects	Blinding of therapists	Blinding of outcome assessment	Dropout rate ≤ 15%	Intention analysis	Between-group statistical comparisons	Point measures and measures of variability	Total
Agnes S. Chan 2013	1	1	1	0	0	0	1	1	1	1	7
Ahmadreza Movahedi 2013	1	0	1	0	0	1	1	1	1	1	7
Fatimah Bahrami 2015	1	0	1	0	1	0	1	1	1	1	7
Janice N. Phung 2019	1	0	1	0	0	0	1	1	1	1	6
Jin-Gui Wang 2020	1	0	1	0	0	0	1	1	1	1	6
Kelong Cai 2020	1	0	1	0	0	0	1	1	1	1	6
Ke-Long Cai 2020	1	0	1	0	0	0	1	1	1	1	6
Mirella Zanobini 2019	1	1	1	0	1	1	1	1	1	1	9
Sabine C Koch2015	1	0	1	0	0	0	0	1	1	1	5
Sixin Yang 2021	1	0	1	0	0	1	1	1	1	1	7
Wenxin Xu 2019	1	0	1	0	0	0	1	1	1	1	6
Mahboubeh G 2018	1	0	1	0	0	0	1	1	1	1	6
Amir H.H 2022	1	0	1	0	0	0	1	1	1	1	6
Supritha Aithal 2021	1	0	1	0	0	0	1	1	1	1	6

1 means that the item is met, and 0 means that the item is not met.

### 3.4. Meta-Analysis

#### 3.4.1. Total scores with respect to social communication disorder

**Figure 2:**
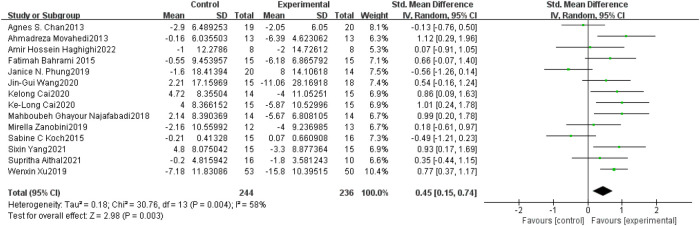
Forest plots of meta-analyses of the effect of physical exercise on social communication disorder in ASD.

The study assessed 14 studies (*N* = 460) that examined the influence of physical exercise on social communication disorder in patients with ASD. The results of the meta-analysis of social communication disorders in the physical exercise group are shown in Figure 2. The heterogeneity test indicated that there was moderate statistical heterogeneity between the studies (*X^2 ^*= 30.76, *I^2 ^*= 58%, *p *= 0.004), thus, the random-effects model was used for the analysis. The meta-analysis showed that the difference was statistically significant, with a pooled effect size SMD = 0.45, 95% CI: 0.15, 0.74, *p *< 0.05. This indicates that physical exercise could effectively improve social communication disorders in autistic patients compared with controls.

#### 3.4.2. Subgroup analysis

Based on the physical exercise intervention period, subgroup analysis was performed. The pooled effect size was measured for a short period (SMD = 0.11, 95% CI: −0.25, 0.47, *p* > 0.05), moderate period (SMD = 0.73, 95% CI: 0.38, 1.08, *p* < 0.001), and long period (SMD = 0.51, 95% CI: 0.24, 0.79, *p* < 0.001). The heterogeneity test showed that *I^2^* = 64%, *p* = 0.04 for a short period, *I^2^* = 0%, *p* = 0.57 for a moderate period, and *I^2^* = 70%, *p* = 0.009 for a long period. Thus, the moderate period and long period were significantly different than the control group, and there was no statistically significant difference between the short period and the control group. Additionally, the moderate period had low heterogeneity, while the short period and long period had moderate heterogeneity (Table 3).

**Table 3:** Meta-analysis results.

Moderating variable		*X^2^* (df)	*I^2^*%	*n*(ES)	ES, 95% CI	*p*
Pooled effect size		30.76 (13)	58	14	SMD = 0.45[0.15, 0.74]	<0.05
The type of exercise	Aerobic exercise	3.02 (4)	0	5	SMD = 0.70[0.37, 1.04]	<0.001
	Mind-body exercise	14.90 (5)	66	6	SMD = 0.08[-0.21, 0.38]	0.59
	Multi-component exercise	2.20 (2)	9	3	SMD = 0.73[0.39, 1.06]	<0.001
Period/week	≤8	8.40 (3)	64	4	SMD = 0.11 [−0.25,0.47]	0.55
	8∼12	2.90 (4)	0	5	SMD = 0.73 [0.38, 1.08]	<0.001
	>12	13.48 (4)	70	5	SMD = 0.51[0.24, 0.79]	<0.001
Frequency (times/week)	Low frequency ≤ 3	11.44 (6)	48	7	SMD = 0.03[−0.37, 0.43]	0.88
	Moderate to high Frequency >5	1.59 (5)	0	6	SMD = 0.84[0.53, 1.14]	<0.001
intervention duration/min	Short duration (≤45)	13.04 (6)	54	7	SMD = 0.44[0.16, 0.73]	<0.05
	Medium duration (45∼60)	0.53 (1)	0	2	SMD = −0.29[−0.76 0.18]	0.23
	Long duration (>60)	3.10 (3)	3	4	SMD = 0.77[0.36,1.18]	<0.001
Organizational form	Group exercise	11.27 (5)	56	6	SMD = 0.51[0.21, 0.82]	<0.001
	Individual exercise	11.76 (3)	74	4	SMD = 0.17[−0.19, 0.53]	0.35
	Individual exercise with Multi-participation	3.76 (3)	20	4	SMD = 0.64[0.33, 0.95]	<0.001
Assessment tools	SRS-2	3.02 (4)	0	5	SMD = 0.70[0.37,1.04]	<0.001
	GARS-2	3.02 (3)	1	4	SMD = 0.76[0.35, 1.17]	<0.001
	Confounding indicators	17.01 (4)	76	5	SMD = 0.02[−0.56, 0.59]	0.95
Age	Preschool-age	3.07 (5)	0	6	SMD = 0.73[0.47, 0.99]	<0.001
	School-age	13.17 (4)	70	5	SMD = 0.45[0.20, 1.10]	0.17
	Adolescence and above	2.38 (2)	16	3	SMD = −0.12[−0.56, 0.33]	0.60

Erikson's Eight Stages of Development was adopted for the following age groups: preschool-age (3–6 years old), school-age (7–12 years old), and adolescence and above (>12 years old).

Subgroup analysis was conducted according to the frequency of physical exercise. The pooled effect size was measured for low (SMD = 0.03, 95% CI: −0.37, 0.43, *p* > 0.05), moderate, and high frequency exercise (SMD = 0.84, 95% CI: 0.53, 1.14, *p* < 0.001). The heterogeneity test showed that *I^2^* = 48%, *p* = 0.08 for low frequency, and *I^2^* = 0%, *p* = 0.90 for moderate and high frequency exercise. The results indicated that there was a statistically significant difference between the moderate and high frequency groups compared with the control group, and there was no statistically significant difference between the low frequency and the control group. Additionally, the low, moderate, and high frequency groups had low heterogeneity (Table 3).

Subgroup analysis was performed based on the physical exercise intervention duration. The pooled effect size was measured for the short (SMD = 0.44, 95% CI: 0.16, 0.73, *p* < 0.05), moderate (SMD = −0.29, 95% CI: −0.76, 0.18, *p* > 0.05), and long duration (SMD = 0.77, 95% CI: 0.36, 1.18, *p* < 0.001). The heterogeneity test showed that *I^2^* = 54%, *p* = 0.04 for the short duration, *I^2^* = 0%, *p* = 0.46 for the moderate duration, and *I^2^* = 3%, *p* = 0.38 for the long duration. The findings indicated that the short duration and long duration groups were significantly different than the control group, and there was no statistically significant difference between the moderate duration and the control group. Additionally, the moderate duration and long duration groups had low heterogeneity, while the short duration had moderate heterogeneity (Table 3).

Subgroup analysis was conducted according to the organizational forms of physical exercise. The pooled effect size was measured for group exercise (SMD = 0.51, 95% CI: 0.21, 0.82, *p* < 0.001), individual exercise (SMD = 0.17, 95% CI: −0.19, 0.53, *p* > 0.05), and individual exercise with multi-participation (SMD = 0.64, 95% CI: 0.33, 0.95, *p* < 0.001). The heterogeneity test showed that *I^2^* = 56%, *p* = 0.05 for group exercise, *I^2^* = 74%, *p* = 0.008 for individual exercise, and *I^2^* = 20%, *P* = 0.29 for individual exercise with multi-participation. The results indicated that the group exercise and individual exercise with multi-participation were significantly different than the control group, while there was no statistically significant difference between the individual exercise group and the control group. Additionally, individual exercise with multi-participation had low heterogeneity, while group exercise and individual exercise had moderate heterogeneity (Table 3).

The study next conducted a subgroup analysis based on the organizational form of the physical exercise intervention. The heterogeneity test for group exercise was statistically significant, with *X^2 ^*= 11.27, *I^2 ^*= 56%, *p *= 0.05, the pooled effect size SMD = 0.51, 95% CI: 0.21, 0.82, *p *< 0.001. The results of the individual exercise were not significant, with *X^2 ^*= 11.76, *I^2 ^*= 74%, *p *= 0.008, the pooled effect size SMD = 0.17, 95% CI: −0.19, 0.53, *p *> 0.05. The results for individual exercise with multi-participation were statistically significant, with *X^2 ^*= 3.76, *I^2 ^*= 20%, *P *= 0.29, and the pooled effect size SMD = 0.64, 95% CI: 0.33, 0.95, *p *< 0.001 (Table 3).

Based on the assessment tools, subgroup analysis was performed. The pooled effect size was measured for SRS−2 (SMD = 0.70, 95% CI: 0.37, 1.04, *p* < 0.001), GARS-2 (SMD = 0.76, 95% CI: 0.35, 1.17, *p* < 0.001), and confounding indicators (SMD = 0.02, 95% CI: −0.56, 0.59, *p* > 0.05). The heterogeneity test showed that *I^2^* = 0%, *p* = 0.55 for SRS-2, *I^2^* = 1%, *p* = 0.39 for GARS-2, and *I^2^* = 76%, *p* = 0.002 for confounding indicators. The findings indicated that the SRS-2 and GARS-2 were significantly different from the control group, and there was no statistically significant difference between the confounding indicators and the control group. Additionally, the SRS-2 and GARS-2 had low heterogeneity, while confounding indicators had moderate heterogeneity (Table 3).

According to the age group, subgroup analysis was conducted. The pooled effect size was measured for participants who were preschool-age (SMD = 0.73, 95% CI: 0.47, 0.99, *p* < 0.001), school-age (SMD = 0.45, 95% CI: −0.20, 1.10, *p* > 0.05), and in adolescence or above (SMD = −0.12, 95% CI: −0.56, 0.60, *p* > 0.05). The heterogeneity test showed that *I^2^* = 0%, *p* = 0.69 for preschool-age, *I^2^* = 70%, *p* = 0.01 for school-age, and *I^2^* = 16%, *p* = 0.30 for adolescent and older participants. The results indicated that the preschool-age and school-age groups were significantly different than the control group, while the adolescent and older group was not significantly different from the control group. Additionally, the preschool-age and adolescent and older groups had low heterogeneity, while the school-age group had moderate heterogeneity (Table 3).

### 3.5. Sensitivity analysis

To explore whether the heterogeneity among studies was caused by a single study, the study next conducted a subgroup analysis based on the organizational form of the physical exercise intervention. The heterogeneity test for group exercise was statistically significant, with X2 = 11.27, I2 = 56%, *p* = 0.05, the pooled effect size SMD = 0.51, 95% CI: 0.21, 0.82, p < 0.001. The results of the individual exercise were not significant, with X2 = 11.76, *I*^2^ = 74%, *p* = 0.008, the pooled effect size SMD = 0.17, 95% CI: −0.19, 0.53, *p* > 0.05. The results for individual exercise with multi-participation were statistically significant, with X2 = 3.76, *I*^2^ = 20%, *P* = 0.29, and the pooled effect size SMD = 0.64, 95% CI: 0.33, 0.95, *p* < 0.001 (Table 3). conducted a sensitivity analysis on the total intervention effect of social communication disorder with moderate heterogeneity and analyzed the pooled effect by excluding individual studies one by one. The pooled effect size of physical exercise on social communication disorders in patients with ASD in all included studies had a SMD = 0.45, 95% CI: 0.15, 0.74, *p *< 0.05, *I^2 ^*= 58%. After excluding any single study, the range of the pooled effect SMD was 0.40 to 0.53, the range of *I^2^* was 45% to 61%, and the *p-values* were all less than 0.05. These results indicated that the data sensitivity was relatively low and that there was no substantial change in the results of the meta-analysis, suggesting that the results had some degree of stability and reliability (Table 4).

**Table 4:** The pooled effect of social communication function after excluding a single study.

Scoring types and included studies	SMD	95% CI	*p* (the pooled effect)	*I^2^* (%)
Agnes S. Chan 2013	0.50	0.20,0.80	*p* < 0.001	55
Ahmadreza Movahedi 2013	0.40	0.10,0.70	*p* < 0.05	58
Amir Hossein Haghighi 2022	0.47	0.16,0.78	*p* < 0.05	60
Fatimah Bahrami 2015	0.43	0.12,0.75	*p* < 0.05	61
Janice N. Phung 2019	0.53	0.26,0.80	*p* < 0.001	45
Jin-Gui Wang 2020	0.44	0.12,0.76	*p* < 0.05	61
Kelong Cai 2020	0.42	0.11,0.73	*p* < 0.05	60
Ke-Long Cai 2020	0.41	0.10,0.71	*p* < 0.05	58
Mahboubeh G. N 2018	0.41	0.10,0.72	*p* < 0.05	59
Mirella Zanobini 2019	0.47	0.15,0.78	*p* < 0.05	60
Sabine C Koch 2015	0.52	0.24,0.80	*p* < 0.001	49
Sixin Yang 2021	0.41	0.10,0.72	*p* < 0.05	59
Supritha Aithal 2021	0.45	0.14,0.77	*p* < 0.05	61
Wenxin Xu 2019	0.41	0.09,0.73	*p* < 0.05	57

### 3.6. Publication bias

**Figure 3:**
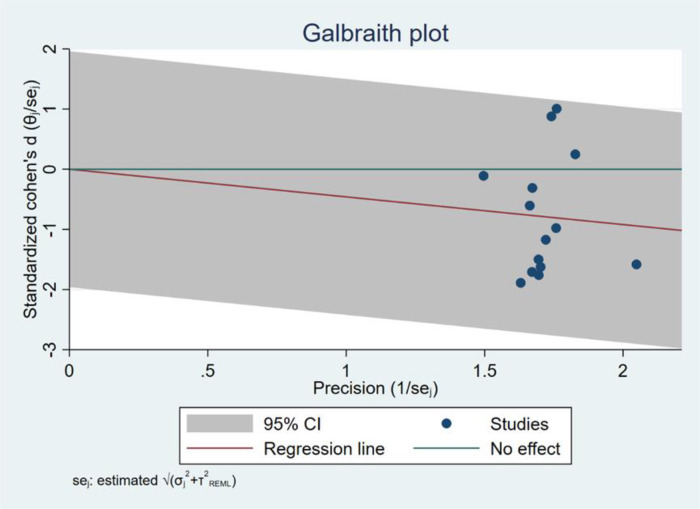
Egger's test results.

Egger's Test showed that there was no significant difference, with *z *= −0.06 (continuity corrected), Pr>|*z*| = 0.949 (continuity corrected) >0.05 (Figure 3). Although there may be some bias because other single studies could not be tested, the total findings indicated that there was no publication bias, and that the results were relatively robust.

## 4. Discussion

The research results showed that physical exercise can improve disordered social communication in patients with ASD, with an effect size of 0.45, which is a medium effect size according to Cohen's evaluation standard (34). This confirms that physical exercise is beneficial to social communication problems in autistic individuals. However, the research found moderate heterogeneity among the included studies, which may be caused by individual differences in physical exercise variables, activity organizational form, age, assessment tools, etc (26)..

The type of exercise is an important variable, given that the exercise ability of autistic patients is a key factor affecting their integration into society. The results showed that while aerobic and multi-component exercise had significant impacts, mind-body exercise did not. This may be because in mind-body exercise, patients are often confined to their personal world, and have little contact with the outside world.

Exercise load, which includes quantity and intensity, is the most crucial concept in sports training. The intervention period, exercise frequency, exercise period, and intensity are concrete manifestations of exercise load, and are significant determinants of how well an exercise intervention works (35, 36). In this study, the intervention period, exercise frequency, and period all had moderating impacts on the intervention effect. From the perspective of the exercise period, an intervention that lasts 8 to 12 weeks is ideal. There may be a downward trend with an extended period, but the research found little difference between the total effect size for the moderate and long intervention period. Additional experiments are required to confirm the effect of exercise intervention length. From the standpoint of exercise duration, more than 60 min of exercise every session is expected to produce greater improvement. This is because, particularly in patients with ASD, too little exercise is not likely to alter the arousal level of the body or the structure and function of the brain. From the perspective of exercise frequency, more than three times a week is expected to produce an effect. Further research is needed to determine whether the effect of exercise conducted more than five times per week is better than that of moderate frequency.

The organizational form of the interventions is important. Patients with ASD often cannot communicate and interact with others in daily life *via* gaze, facial expressions, body posture, etc. It can be difficult to develop high quality friendships because of reduced sharing and cooperation in peer communication (37, 38). The subgroup analysis of the organizational form of the physical exercise revealed that group exercise produced significant intervention effects, while individual exercise did not. Furthermore, the research found that the intervention effect was better for individual exercise with multi-participation. This may be because teachers provide more attention to each participant during individual exercise, and are able to be more patient when teaching basic skills. After mastering individual exercise skills, participants could better participate in group activities, such that physical exercise games or group activities could be carried out more successfully. Interactions between patients were increased during exercise, which is conducive to the further development of social communication (31, 39). Complex and diverse forms of group interventions can elicit participant interest, interactivity, ecological effects, etc. Autistic patients can benefit from multi-role interactive group exercise, which provides a rich social environment (40). The collective classroom environment also provides patients with information about basic social etiquette, such as teacher-student greetings, peer greetings, parent-child interactions, waiting in line, etc., which can offer a good foundation for integration into campus and society (41). Generally speaking, for autistic patients, more peer communication and interaction during physical exercise can promote the improvement of core symptoms.

For those with ASD, early intervention is crucial (42). This study demonstrated that as age increased, the intervention effect had a downward trend. The intervention effect was observed in preschool-age children, but was not significant after adolescence. Therefore, early screening and early intervention are needed, as interventions in childhood can effectively improve symptoms (43). As the complexity of language, social skills, and motor skills increases with age, skill deficits in individuals with ASD may expand in the future. This could explain why the improvement in each functional area decreases with age (44).

The intervention effect of physical exercise on social communication in autistic patients is influenced by the assessment tools used (45, 46). Of those assessed in the present study, GARS-2 had the largest effect size (SMD = 0.76), SRS-2 had a moderate effect size (SMD = 0.70), and there was no significant difference between the experimental group and the control group in terms of confounding indicators (*p *> 0.05). The evaluation of ASD symptoms includes a large number of components, which leads to differences in sensitivity and specificity. Moreover, the sensitivity and specificity of tools for screening and diagnosing ASD can be influenced by the judgment criteria, applicable objects, and screening thresholds (47). Although the DSM-5 is the most current diagnostic standard, several scales used in the included studies were based on the DSM-4 and earlier standards. Furthermore, some of the items in the scales did not apply to all the subjects (48). The threshold of a scale will impact its sensitivity and specificity, and the screening threshold of different scales may not be suitable for all ASD screening procedures (48).

Physical exercise can provide a social environment with a unique opportunity for social learning and practicing social skills such as observation, imitation, and emotional regulation outside of a clinical environment (49). Previous studies on children and adolescents with ASDs have shown that physical activities such as karate, swimming, and basketball are associated with improved social interaction, communication, stereotyped behavior, motor skills, motor coordination, cardiovascular health, and quality of life (13, 14, 26, 28). Physical exercise may change the motor abilities of ASD patients, thereby promoting the improvement of their social skills (10, 15). The relationship between physical exercise and social function seems to be bidirectional. Children with social communication disorder are less willing to participate in physical exercise, which may further reduce their opportunity to practice social skills, and may limit the acquisition of certain sports skills that depend on interactions with others. Perceptual motor training enhances the function and cognitive performance of the nervous system by promoting neural plasticity, creating new synaptic structures, and reducing cognitive impairments (14, 50). Exercise-induced increases in neural, physiological, and developmental function lead to improved attention and performance, which increases the attentional function of children with autism (51).

Social interaction is an important aspect of many physical exercise interventions, which include communication, physical contact, eye contact, and language. These are important factors in promoting social interaction skills. Thus, physical exercise can play an important role in promoting social interaction and social skills (52). This is because people can communicate in a relaxed and enjoyable environment through exercise, *via* speech, gestures, and actions. Biological motion is an important carrier of social information and can provide relevant information for social interaction. The visual and perceptual processing of biological motion by individuals is of great value for the smooth completion of daily activities, especially for adaptive social behavior and nonverbal communication (53). During activities that involve social interaction, companion communication, physical contact, eye contact, and language are essential and can help children interact with their peers, reduce fear of the environment, and improve participation and cooperation with their peers. Improvements in all these aspects may enhance social interaction skills in children with ASD (54, 55).

This study found that physical exercise had a significant effect in improving social interaction difficulties in patients with ASD. The best exercise effects were achieved through early interventions, multi-component exercise, and exercise with moderate periods, high frequency, long duration, and group participation. This provides a new intervention approach for improving symptoms in patients with ASDs. Although the research recommend the use of physical exercise as an intervention for ASD, more research is needed to determine the best exercise programs for these patients. To accurately understand the role of different physical exercise programs in the treatment of children with ASD, more experimental research involving ASD patients is needed, with a focus on global results. Future studies can build on this meta-analysis by reporting complete data (between-group effects, confidence intervals, and effect sizes), studying similar populations, using more specific exercise variables, and measuring the same or related global outcomes.

This study has several limitations: (1) The limited number of included studies only allowed for a rough estimate of the effects of exercise time, frequency, period, and age, and more experiments are needed to explore the effects of exercise load. The research only analyzed the optimal “dose” according to a single factor, and so the combined effects of multiple factors were not accurately reflected in the findings. (2) Physical exercise interventions are difficult to administer in a double-blind experimental setting, and many problems that exist during the intervention process, due to the special nature of ASD patients, can affect experimental results. (3) Most of the outcome indicators included in this study were judged by questionnaires, which are easily influenced by subjective factors. In the future, more objective evaluation tools are needed.

## 5. Conclusions

Physical exercise has a significant improvement effect on the social communication difficulties in individuals with ASD. Early intervention can lead to greater improvements in social communication and lay a foundation for better social integration in the future. With regards to social communication difficulties, exercise programs that are multi-component, of moderate duration, moderate-high frequency, long-term, and that involve multiple people are most effective in improving ASD symptoms. This intervention strategy merits further research.

## Data Availability

The original contributions presented in the study are included in the article/Supplementary Material, further inquiries can be directed to the corresponding author.
